# Expression of the *NRF2* Target Gene *NQO1* Is Enhanced in Mononuclear Cells in Human Chronic Kidney Disease

**DOI:** 10.1155/2017/9091879

**Published:** 2017-07-13

**Authors:** Jianlin Shen, Marianne Rasmussen, Qi-Rong Dong, Martin Tepel, Alexandra Scholze

**Affiliations:** ^1^Department of Orthopedics, The Second Affiliated Hospital of Soochow University, Suzhou, Jiangsu, China; ^2^Institute of Molecular Medicine, Cardiovascular and Renal Research, University of Southern Denmark, Odense, Denmark; ^3^Department of Nephrology, Odense University Hospital, Odense, Denmark; ^4^Institute of Clinical Research, University of Southern Denmark, Odense, Denmark

## Abstract

Reduced nuclear factor erythroid 2-related factor 2 (*NRF2*) pathway activity was reported in models of chronic kidney disease (CKD). Pharmacological activation of *NRF2* is supposed to improve renal function, but data concerning the *NRF2* activity in human CKD are lacking. We investigated the *NRF2* target NAD(P)H:quinone oxidoreductase 1 (*NQO1*) as a readout parameter for *NRF2* activity in monocytes of CKD patients (*n* = 63) compared to those of healthy controls (*n* = 16). The *NQO1* gene expression was quantified using real-time PCR and the protein content by in-cell Western assays. We found a 3-4-fold increase in *NQO1* gene expression in CKD 1–5 (*n* = 29; 3.5 for *NQO1*/ribosomal protein L41; *p* < 0.001). This was accompanied by a 1.1-fold increase in *NQO1* protein (*p* = 0.06). Cardiovascular disease prevalence was higher in CKD 1–5 patients with higher compared to those with lower *NQO1* gene expression (*p* = 0.02). In advanced uremia, in dialysis patients (*n* = 34), *NQO1* gene expression was less robustly upregulated than that in CKD 1–5, while *NQO1* protein was not upregulated. We conclude that in mononuclear cells of CKD patients, the *NRF2* pathway is activated by coexisting pathogenic mechanisms, but in advanced uremia, the effectiveness of this upregulation is reduced. Both processes could interfere with pharmacological *NRF2* activation.

## 1. Introduction

The transcription factor *NRF2* is a master transcriptional regulator of cellular response to oxidative and electrophilic stress. It activates a multitude of cellular defense processes through induction of its target genes, including drug-metabolizing and antioxidant enzyme genes [1]. Oxidative or electrophilic stress can activate the *NRF2* pathway by an interaction with the cytoplasmic complex between Kelch-like ECH-associated protein 1 (*KEAP1*) and *NRF2*. As a result, *NRF2* can escape ubiquitination and proteasomal degradation, accumulate, and translocate to the nucleus. It forms heterodimers with small Maf proteins, binds to antioxidant/electrophilic response elements, and thereby finally induces target gene expression [1, 2].

Cytosolic *NQO1* is a conserved target gene of *NRF2* and can serve to monitor the activity of the *NRF2* pathway [2]. During its clinical development, the *NRF2* activator bardoxolone methyl was investigated in a phase 1 clinical trial, in patients with advanced malignancies. In this trial, the *NQO1* gene expression levels in peripheral blood mononuclear cells (PBMCs) were shown to be indicative of *NRF2* pathway activation by the substance [3].

In animal models of renal diseases, impaired activity of the *NRF2* pathway and downregulation of the *NQO1* gene expression are major findings, both in renal and in nonrenal cells [2, 4, 5]. Furthermore, it was shown in animal models that activation of the *NRF2* pathway and increased gene transcription, including those of *NQO1*, attenuated kidney injury [6–8]. Therefore, there is a strong interest in the therapeutic potential of *NRF2* activators in kidney disease [9]. A phase 2b clinical trial with the *NRF2* activator bardoxolone methyl in CKD stages 3b and 4 demonstrated an improvement in estimated glomerular filtration rate (eGFR) but suggested adverse effects, for example, on liver tissue [10]. A phase 3 clinical trial in CKD stage 4 patients (BEACON) with the same substance also showed an increased eGFR but had to be terminated because of a higher rate of cardiovascular events in the treatment group [11]. Currently, one more phase 2 clinical trial with bardoxolone methyl in CKD patients is recruiting participants (NCT02316821). In the publication of the so far largest clinical trial of bardoxolone methyl, the authors refer to the impairment of *NRF2* activity-dependent gene transcription that was reported in animal models of CKD as one rational for the clinical application of the substance in CKD [11]. Accordingly, in our study, we investigated the gene expression of *NQO1* as a parameter of *NRF2*-dependent gene transcription in human CKD. We present a systematic analysis of this drug target on the level of gene expression and protein content in CKD patients with and without renal replacement therapy. The BEACON trial was terminated because of a higher rate of cardiovascular events in the intervention group [11]. A post hoc analysis by the manufacturer of the substance identified prior hospitalization for heart failure as a risk factor for heart failure experienced during bardoxolone treatment [12]. Therefore, we investigated a relation between the *NQO1* gene expression as a measure for *NRF2* pathway activity and cardiovascular disease (CVD) prevalence in patients with non-dialysis-dependent CKD.

Since systemic *NRF2* activators target the *NRF2* pathway systemically and nonrenal adverse events were reported, both nonrenal and renal cells of CKD patients require investigation.

We therefore quantified the *NQO1* gene expression as a readout parameter for *NRF2* pathway activity in monocytes of patients with CKD with and without dialysis therapy and compared it to those of healthy control subjects. In parallel, *NQO1* protein content was quantified in cells of the same subjects.

## 2. Subjects and Methods

### 2.1. Study Subjects

We prospectively enrolled 63 consecutive patients with CKD from the outpatient clinics of the Department of Nephrology, Odense University Hospital, and 16 healthy control subjects. Among the group of CKD patients, 34 were undergoing dialysis therapy (hemodialysis, *n* = 33; peritoneal dialysis, *n* = 1). The study was approved by the regional ethics committee and written informed consent was obtained. Hemodialysis patients were dialyzed with biocompatible membranes, three times per week, for 4-4.5 hours. Healthy control subjects were free from chronic or acute disease. Study participants were 30 years or older. CKD patients had verified CKD according to the 2012 Clinical Practice Guideline for the Evaluation and Management of Chronic Kidney Disease, and patients with dialysis therapy had been in dialysis treatment for at least 3 months [13]. Exclusion criteria included pregnancy or breastfeeding, acute illness leading to hospital admission, and functioning renal allograft.

### 2.2. Collection of Clinical Data and Blood Samples

Clinical data were obtained by physical examination, medical history taking, and electronic medical records. This included the record of age, sex, height, weight, smoking status, medical history, and use of medications. Venous blood samples were drawn in the morning from study participants. Samples from hemodialysis patients were obtained before the start of hemodialysis sessions. Plasma and serum were separated and stored at −80°C. The eGFR was calculated using the 2009 CKD-EPI creatinine equation, and CKD stages were determined based on eGFR categories according to the KDIGO 2012 guideline [13].

### 2.3. Isolation of Circulating Monocytes

We isolated PBMCs by density gradient centrifugation (Histopaque-1077, Sigma-Aldrich, Denmark). Monocytes then were isolated from PBMCs using anti-CD14-coated superparamagnetic polystyrene beads (Fisher Scientific, Denmark) according to the manufacturer's protocol.

### 2.4. RNA Isolation, Reverse Transcription, and Quantitative Real-Time PCR (RT-qPCR)

Total RNA was isolated from monocytes using an RNeasy Mini kit (Qiagen, Denmark) according to the protocol described by the manufacturer. cDNA was synthesized from 200 ng of total RNA by reverse transcription using a Transcriptor First-Strand cDNA Synthesis Kit (Roche, Denmark). The amplification of genes was performed by quantitative real-time PCR using SYBR Green (FastStart Essential DNA Green Master, Roche, Denmark). The PCR conditions using a LightCycler96 Instrument (Roche, Denmark) were as follows: 95°C for 600 s and 50 cycles of 95°C for 10 s, 64°C (for *NQO1*, beta-actin (*ACTB*), and ribosomal protein L41 (*RPL41*)) or 60°C (for TATA-box binding protein (*TBP*)) for 10 s, and 72°C for 10 s. The primers used were in Table 1.

To ensure a high reliability of the gene expression results in our study, we tested a group of available reference genes in the cellular material of our study. We then employed the “NormFinder” algorithm (http://moma.dk/normfinder-software; [14]). The reference genes with the highest expression stability in our sample material were *RPL41*, *ACTB*, and *TBP* with stability values (SE) of 0.12 (0.06), 0.18 (0.06), and 0.22 (0.05), respectively. We therefore used these three genes for the relative quantification of *NQO1* gene expression. The ratio was calculated as follows: ratio = 2^(Cq reference gene)^/2^(Cq *NQO1*)^.

PCR products were size-fractionated on agarose gels for product length control.

In the PCRs, water controls, no-template controls, and no-RT controls were included. A melting curve analysis was performed for each sample to ensure product homogeneity. All measurements were performed in duplicate.

### 2.5. SDS-PAGE and Western Blotting

Protein was extracted from monocytes using an extraction reagent for mammalian cells including a protease inhibitor cocktail (cOmplete Lysis-M, pH 7.6, Roche, Denmark). Proteins were separated by 10% sodium-dodecyl-sulfate polyacrylamide gel electrophoresis (10% RunBlue SDS gel, Expedeon, UK) at 120 V for 45 minutes and transferred to polyvinylidendifluoride membranes at 100 V for 75 minutes (Immun-Blot LF PVDF, Bio-Rad, USA). Membranes were blocked with blocking buffer (Superblock, Thermo Fisher Scientific, USA) for 1 hour at room temperature and incubated with the primary antibodies rabbit anti-human *NQO1* (ab34173, Abcam, UK) or rabbit anti-human *ACTB* (sc-130656, Santa Cruz Biotechnology, Germany). After washing with tris(hydroxymethyl)aminomethane-buffered saline, the membranes were incubated with the fluorescence-labeled secondary antibody F(ab′)_2_-goat anti-rabbit IgG (H+L) Alexa Fluor 488 (Fisher Scientific, Denmark). Imaging was performed using a Carestream Imager 4000pro (Fisher Scientific, Denmark) at 535 nm emission with an excitation wavelength of 470 nm.

### 2.6. Quantitative In-Cell Western Assay

To quantify the *NQO1* protein content in circulating monocytes, in-cell Western assays were performed as recently described by our group [15, 16]. For that purpose, monocytes were fixed with formaldehyde and permeabilized using Triton X-100 in 96-well plates. Cells were blocked using 1% bovine serum albumin overnight at 4°C, then incubated with the primary antibodies rabbit anti-human *NQO1* (ab34173, Abcam, UK) or rabbit anti-human *ACTB* (sc-130656, Santa Cruz Biotechnology, Germany). After washing, cells were incubated with the fluorescence-labeled secondary antibody F(ab′)_2_-goat anti-rabbit IgG (H+L) Alexa Fluor 488 (Fisher Scientific, Denmark). All measurements were performed in triplicate, and the *NQO1* protein content was analyzed relative to the *ACTB* protein content as an internal reference. Imaging was performed using an EnSpire Multimode Plate Reader (PerkinElmer, Denmark) at 520 nm emission with an excitation wavelength of 490 nm.

## 3. Statistics

Continuous variables are given as median and interquartile range, and categorical variables are given as counts and percentages. Groups were compared using Kruskal-Wallis test with Dunn's posttest or Mann–Whitney test. Differences in categorical variables between groups were analyzed by *Χ*^2^ test (GraphPad prism software, version 5.0, GraphPad Software, San Diego, CA). All statistical tests were two-sided and a two-sided value of *p* < 0.05 was considered statistically significant.

## 4. Results

Population characteristics of study subjects are shown in Table 2.

**Figure 1:**
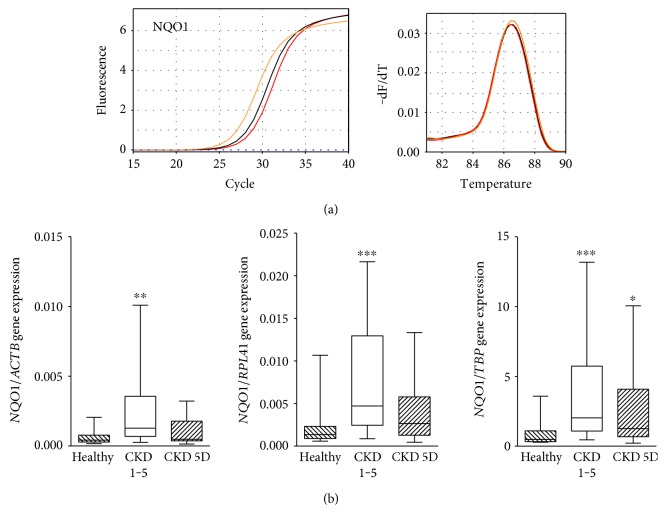
*NQO1* gene expression. (a) Amplification curves and melting curves for *NQO1* in monocytes from a patient with CKD 4 (CKD, orange), a hemodialysis patient (CKD 5D, black), and a healthy subject (red). (b) Box and whisker plots (whiskers, minimum to maximum) showing summary data of the *NQO1* gene expression in healthy subjects (*n* = 16), CKD 1–5 patients (*n* = 29), and CKD 5D patients (*n* = 34) normalized to *ACTB*, *RPL41*, and *TBP*. Comparison by Dunn's posttest. ^∗^*p* < 0.05; ^∗∗^*p* < 0.01; ^∗∗∗^*p* < 0.001.

The *NQO1* gene expression was significantly higher in monocytes from patients with CKD 1–5 (Kruskal-Wallis, *p* = 0.004 for *NQO1*/*ACTB*; *p* < 0.001 for *NQO1*/*RPL41*; and *p* < 0.001 for *NQO1*/*TBP*; Figures 1(a) and 1 (b)). Compared to healthy controls, patients with CKD 1–5 showed a 3-4-fold increase in the *NQO1* gene expression (3.1 for *NQO1*/*ACTB*, 3.5 for *NQO1*/*RPL41*, and 4.2 for *NQO1*/*TBP*). In CKD 5 patients undergoing dialysis therapy (CKD 5D), median *NQO1* gene expression was numerically higher than that in healthy controls but this was significant only for the *NQO1*/*TBP* ratio (a 2.6-fold increase for *NQO1*/*TBP*, *p* < 0.05, Figure 1(b)). The relation between the *NQO1* gene expression level and CKD stage followed approximately a bell shape. This is shown in the Supplementary Figure 1 available online at https://doi.org/10.1155/2017/9091879 representing the distribution of the *NQO1* gene expression at the different CKD stages including advanced CKD with hemodialysis therapy (Kruskal-Wallis, *p* = 0.005).

**Figure 2:**
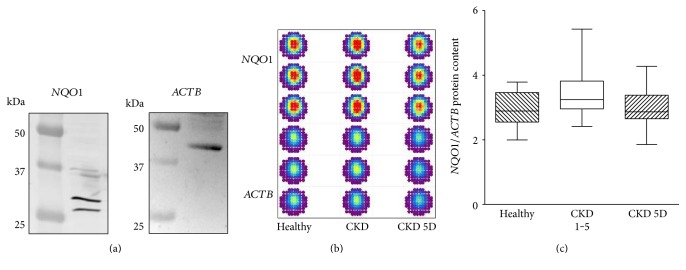
*NQO1* protein content. (a) To show the effective detection of *NQO1* and *ACTB* protein by antibodies in the cell material used in our study, we performed immunoblot analyses with cells obtained from healthy control subjects. Immunoblots of *NQO1* (expected size 26/27 kDa and 31 kDa) and *ACTB* (expected size 42 kDa) in monocytes are shown. (b) Pseudocolored fluorescence intensities of in-cell Western assays for the quantification of the *NQO1* protein content relative to the *ACTB* protein content in monocytes from a healthy subject, a patient with CKD, and a patient with CKD 5D. Measurements were always performed in triplicate. (c) Box and whisker plots (whiskers, minimum to maximum) showing summary data of *NQO1* protein in healthy subjects (*n* = 13), CKD 1–5 patients (*n* = 23), and CKD 5D patients (*n* = 29) normalized to *ACTB*. *p* = 0.07 by Kruskal-Wallis test.

We also quantified the *NQO1* protein content in cells of the identical blood samples used for the gene expression analyses described above. First, we proved that in the cell material used for our study, the antibodies effectively detected *NQO1* and *ACTB* proteins. Immunoblot analyses showed staining of protein bands at the expected sizes (Figure 2(a)).  Figure 2(b) shows a representative example of an in-cell Western assay used for the relative quantification of the NQO1 protein content in monocytes. Measurements were performed in triplicate for each subject.  Figure 2(c) depicts the summary data for all in-cell Western analyses. The *NOQ1* protein content showed a 1.1-fold increase in CKD 1–5 patients compared to healthy controls (Mann–Whitney, *p* = 0.06). In CKD 5D, the *NQO1* protein content was not different from control subjects (Mann–Whitney, *p* = 0.72).

Furthermore, since cardiovascular events led to the early termination of a large *NRF2* activator trial in non-hemodialysis-dependent CKD, we investigated the prevalence of CVD in our study. For the analysis of the *NQO1* expression and prevalence of CVD in non-hemodialysis-dependent CKD stages 1–5, we grouped the patients according to their *NQO1* gene expression below or above the median and compared the frequency of CVD between them. When we analyzed the overall prevalence of CVD (myocardial infarction/coronary artery disease, heart failure, cerebrovascular disease, and peripheral artery disease), it was 2 out of 14 in the patient group below median gene expression whereas in the group above median *NQO1* gene expression, the prevalence of CVD was 8 out of 14 (*Χ*^2^ test, *p* = 0.02). The only common underlying single disease type in our patient group was cardiac insufficiency (1 out of 14 in the below median group versus 7 out of 14 in the above median group; *Χ*^2^ test, *p* = 0.18).

## 5. Discussion

This study showed for mononuclear cells of CKD patients (i) increased gene expression of the *NRF2* target *NQO1* in CKD 1–5, (ii) compared to CKD 1–5 a less robust increase in the *NQO1* gene expression in CKD 5D, (iii) slightly increased *NQO1* protein content in CKD 1–5, and (iv) higher prevalence of CVD among CKD 1–5 patients with a higher *NQO1* gene expression.

In our study, we investigated monocytes from peripheral blood of CKD patients. The monocyte-macrophage lineage is of special importance in CKD. On the one hand, they are involved in kidney injury and fibrosis [17, 18]. On the other hand, monocytes and monocyte-derived macrophages are involved in the pathogenesis of atherosclerosis and vascular disease in CKD [19–22]. The latter is important with respect to our finding of higher CVD prevalence in CKD patients with an *NQO1* gene expression above the median in monocytes.

Why did we choose *NQO1* and in particular the *NQO1* gene expression as a readout parameter for *NRF2* pathway activity and activation in kidney disease? Several lines of evidence support our approach. First, *NQO1* belongs to the conserved *NRF2* target genes, and in a recent integrated transcriptomic and proteomic analysis of *NRF2* function, *NQO1* was confirmed as a robust marker for *NRF2* activity [2, 23]. Second, reduced expression of *NQO1* was shown in several kidney disease models, also in nonrenal cells, and upregulation of *NQO1* was repeatedly shown in *NRF2*-dependent kidney protection, again including nonrenal cells [4–8, 24]. Third, it has been shown that the *NQO1* expression is stimulated by the *NRF2* activators CDDO (1-[2-cyano-3-,12-dioxooleana-1,9(11)-dien-28-oyl]imidazolide), dh404 (2-cyano-3,12-dioxooleana-1,9-dien-28-oic acid-9,11-dihydro-trifluoroethyl amide), and bardoxolone methyl and that this effect is abrogated in *NRF2*^−/−^ mice [5, 8, 23]. Notably, bardoxolone methyl stimulates the *NQO1* gene expression also in PBMCs [3].

Some consideration should be given to the effect size of the *NQO1* increase in our study. With respect to the gene expression of *NQO1*, we confirmed a 3-4-fold increase in *NQO1* mRNA in CKD 1–5 patients compared to healthy control subjects using three different housekeeping genes for the relative quantification. A 5.6-fold increase in *NQO1* mRNA was reported to be present in PBMCs from cancer patients after 3 weeks of treatment with bardoxolone methyl [3]. In T cell-specific *KEAP1*-deficient mice, T cells showed an 8-fold relative change in *NQO1* mRNA [7]. In a mouse model of ischemia-reperfusion injury- (IRI-) induced kidney damage, the maximal response of *NQO1* mRNA to IRI was an ~1.8-fold increase in wild-type mice and a 5-fold increase in *KEAP1*-knockdown mice [8]. We therefore conclude that the difference in *NQO1* mRNA level that we observed in our study is of relevant magnitude and points to an activation of the *NRF2* pathway in CKD patients per se.

On the protein level, the *NQO1* protein content in CKD 1–5 patients was not significantly increased to 1.1-fold compared to that in control subjects in our study. Similarly, the treatment of mice with bardoxolone methyl resulted in an ~1.3-fold increase in *NQO1* protein level [23]. Treatment of 5/6 nephrectomy rats with dh404 resulted in an ~3.3-fold increase in *NQO1* protein in the colon [5]. However, the increase in the *NQO1* protein content that we observed in CKD 1–5 needs further investigation.

Effects on gene expression and protein content in CKD are mediated by a multitude of mechanisms. The observed increase in the *NQO1* gene expression in our study might be a response to NRF2-stimulating conditions prevalent in patients with CKD, like oxidative stress or lipopolysaccharide-induced inflammation [25, 26]. Patients with coexisting CVD, which is also associated with oxidative stress, might be especially affected [27]. This is supported by the higher CVD prevalence in CKD 1–5 patients with a higher *NQO1* gene expression that we observed. In addition, low concentrations of the uremic toxin methylglyoxal were shown to increase the *NQO1* mRNA concentration [28].

We also observed that the increase in the *NQO1* gene expression in CKD 5D patients was less pronounced compared to that in CKD 1–5 patients. This is in line with a depressive effect of high concentrations of uremic toxins on the *NRF2* pathway. This effect might be more pronounced in this patient group with advanced uremia. For example, the uremic toxin indoxyl sulfate was shown to downregulate *NRF2* mRNA and *NRF2* protein content [24].

The protein content of *NQO1* appeared slightly upregulated in CKD 1–5 patients and not upregulated in long-term uremic CKD 5D patients. Such discrepancy between gene expression and protein content was already demonstrated by our group for another cytosolic antioxidant protein that also is under the transcriptional regulation of an antioxidant response element—superoxide dismutase type 1 [15].

Our study adds important information to the discussion about the putative use of *NRF2* activators in CKD. *NRF2* activators serve to upregulate the expression of *NRF2* target genes like *NQO1*. However, our study showed that the *NQO1* gene expression was already significantly upregulated in monocytes, which points to a relevant stimulation of the *NRF2* pathway in these cells in CKD patients. Therefore, as the systemic application of *NRF2* activators affects different cell and tissue types, the *NRF2* pathway in kidney disease needs to be investigated in different human cells and tissues. This is of relevance as the overactivation of a potent transcription factor like *NRF2* was suggested as potentially deleterious for the cardiovascular system [27]. Therefore, a preexisting activation of the *NRF2* system in CKD patients especially with CVD, as suggested by our results, could be seen in line with the increased rate of cardiovascular events in the BEACON trial [11]. Moreover, an additional depressive effect of CKD on antioxidant enzymes, especially in advanced uremic conditions, could reduce effectiveness of *NRF2* activation.

Taken together, we showed a relevant upregulation in gene expression of the *NRF2* target *NQO1* in patients with CKD 1–5 together with a slight increase in the *NQO1* protein content in monocytes from these patients. Moreover, we found that in more pronounced uremia (CKD 5D), the *NQO1* gene expression was less upregulated than that in CKD 1–5 and *NQO1* protein content was not increased. We conclude that in CKD patients, *NRF2* activation is modulated through influence on both gene expression and protein content of *NRF2* targets in a complex way.

## Supplementary Material

Supplementary figure 1 NQO1 gene expression and stage of CKD. Box-and-whisker plots (whiskers, minimum to maximum) showing summary data of the NQO1 gene expression relative to RPL41 in healthy subjects (n=16), CKD 1-3a (n=6), CKD 3b (n=8), CKD 4 (n=12), CKD 5 (n=3) and CKD 5 patients with dialysis treatment(CKD 5D; n=34). ٭p<0.05 by Dunn's post test.

## Figures and Tables

**Table 1:** 

Name of gene product	Forward primer	Expected PCR product length (bp)
	Reverse primer	
*NQO1* NM_000903.2	5′-CTGCCATCATGCCTGACTAA-3′ 5′-TGCAGATGTACGGTGTGGAT-3′	216
*ACTB* NM_001101.3	5′-GGACTTCGAGCAAGAGATGG-3′ 5′-AGCACTGTGTTGGCGTACAG-3′	234
*RPL41* NM_021104.1	5′-AAGATGAGGCAGAGGTCC-3′ 5′-TCCAGAATGTCACAGGTCCA-3′	248
*TBP* NM_003194.4	5′-TGCACAGGAGCCAAGAGTGAA-3′ 5′-CACATCACAGCTCCCCACCA-3′	132

**Table 2:** Clinical and demographical population characteristics.

	Healthy (*n* = 16)	CKD 1–5 (*n* = 29)	CKD 5D (*n* = 34)
Age, years	38 (33–48)	68 (58–77)	67 (55–76)
Men, *n* (%)	9 (56)	19 (66)	23 (68)
BMI, kg/m^2^	24 (22–26)	30 (24–37)^a^	24 (22–28)^b^
Smoking, *n* (%)	2 (13)	3 (10)	6 (18)
Kidney disease, underlying cause, *n* (%)	None		
Glomerulonephritis		8 (28)	5 (15)
Hypertensive nephropathy		3 (10)	5 (15)
Interstitial nephritis		2 (7)	4 (12)
Diabetic nephropathy		1 (3)	2 (6)
Hereditary kidney disease		1 (3)	2 (6)
Other/unknown		14 (48)	16 (47)
Comorbidities, *n* (%)	None
Hypertension		28 (97)	26 (76)
Diabetes		8 (28)	8 (24)
CVD			
Myocardial infarction, coronary artery disease		6 (21)	10 (29)
Heart failure		8 (28)	16 (47)
Cerebrovascular disease		4 (14)	6 (18)
Peripheral artery disease		1 (3)	9 (26)
Medications	None
AT receptor antagonist, ACE inhibitor		22 (76)	16 (47)
Beta blocker		9 (31)	15 (44)
Calcium channel inhibitors		13 (45)	9 (26)
Platelet aggregation inhibitor		6 (21)	6 (18)
Diuretic		19 (66)	11 (32)
Erythropoietin analog		6 (21)	29 (85)
Coumarin derivatives		5 (17)	6 (18)
eGFR, mL/min/1.73m^2^	n.d.	28 (17–43)	n.a.
Time on dialysis, months	n.a.	n.a.	22 (11–49)
CRP, mg/L	n.d.	5.3 (2.3–10.8)^c^	3.0 (1.4–10.5)^d^
Albumin, g/L	n.d.	38 (35–41)^e^	38 (36–40)

Values are given as median (25%–75% percentile) or number (percentage). BMI: body mass index; AT: angiotensin; ACE: angiotensin converting enzyme; CRP: C-reactive protein; n.d.: not done; n.a.: not applicable. ^a^*n* = 27; ^b^*n* = 33; ^c^*n* = 28; ^d^*n* = 33; ^e^*n* = 28.
