# Supplementary material for: Expression of the NRF2 Target Gene NQO1 Is Enhanced in Mononuclear Cells in Human Chronic Kidney Disease
Source: Oxid Med Cell Longev. 2017 Jul 13;2017:9091879. doi: 10.1155/2017/9091879 (PMC5530440; doi:10.1155/2017/9091879)
Supplement: Supplementary file 1 — Supplementary figure 1 NQO1 gene expression and stage of CKD. Box-and-whisker plots (whiskers, minimum to maximum) showing summary data of the NQO1 gene expression relative to RPL41 in healthy subjects (n=16), CKD 1-3a (n=6), CKD 3b (n=8), CKD 4 (n=12), CKD 5 (n=3) and CKD 5 patients with dialysis treatment(CKD 5D; n=34). ٭p<0.05 by Dunn's post test. [file 9091879.f1.pdf]

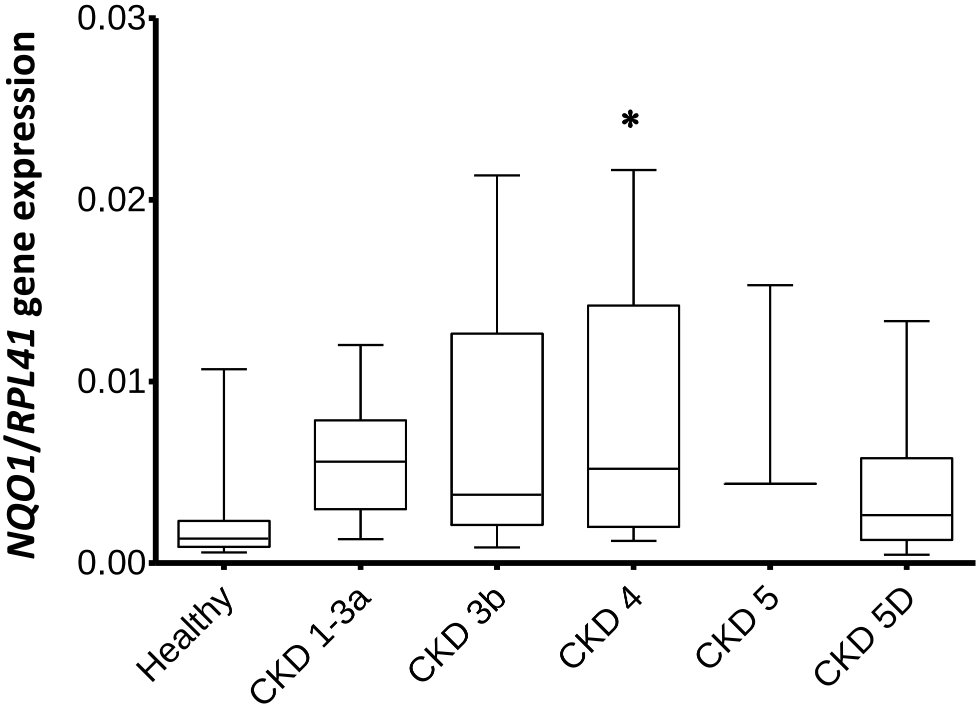

Supplementary figure 1 Shen et al.

Figure legend

Supplementary figure 1 *NQO1* gene expression and stage of CKD.

Box-and-whisker plots (whiskers, minimum to maximum) showing summary data of the *NQO1* gene expression relative to *RPL41* in healthy subjects (n=16), CKD 1-3a (n=6), CKD 3b (n=8), CKD 4 (n=12), CKD 5 (n=3) and CKD 5 patients with dialysis treatment (CKD 5D; n=34). ∗ $p<0.05$ by Dunn’s post test.
